# Applying FAIR Principles to Plant Phenotypic Data Management in GnpIS

**DOI:** 10.34133/2019/1671403

**Published:** 2019-04-30

**Authors:** C. Pommier, C. Michotey, G. Cornut, P. Roumet, E. Duchêne, R. Flores, A. Lebreton, M. Alaux, S. Durand, E. Kimmel, T. Letellier, G. Merceron, M. Laine, C. Guerche, M. Loaec, D. Steinbach, M. A. Laporte, E. Arnaud, H. Quesneville, A. F. Adam-Blondon

**Affiliations:** ^1^URGI, INRA, Université Paris-Saclay, 78026 Versailles, France; ^2^AGAP, Univ Montpellier, CIRAD, INRA, Montpellier SupAgro, Montpellier, France; ^3^UMR SVQV, 28 rue de Herrlisheim, B.P. 20507, 68021 Colmar, France; ^4^Bioversity International, parc Scientifique Agropolis II, 34397 Montpellier cedex 5, France

## Abstract

GnpIS is a data repository for plant phenomics that stores whole field and greenhouse experimental data including environment measures. It allows long-term access to datasets following the FAIR principles: Findable, Accessible, Interoperable, and Reusable, by using a flexible and original approach. It is based on a generic and ontology driven data model and an innovative software architecture that uncouples data integration, storage, and querying. It takes advantage of international standards including the Crop Ontology, MIAPPE, and the Breeding API. GnpIS allows handling data for a wide range of species and experiment types, including multiannual perennial plants experimental network or annual plant trials with either raw data,* i.e.,* direct measures, or computed traits. It also ensures the integration and the interoperability among phenotyping datasets and with genotyping data. This is achieved through a careful curation and annotation of the key resources conducted in close collaboration with the communities providing data. Our repository follows the Open Science data publication principles by ensuring citability of each dataset. Finally, GnpIS compliance with international standards enables its interoperability with other data repositories hence allowing data links between phenotype and other data types. GnpIS can therefore contribute to emerging international federations of information systems.

## 1. Introduction

Plant phenotyping regroups all the observations and measures that can be made on a precisely identified plant material in a characterized environment. This very general definition of phenomics [1] includes diverse types of properties and variables measured at different physical [2] and temporal scales, ranging from field observation of plant populations to molecular cell characterizations, including for some research community metabolomics or gene expression. The acquisition of these data is conducted in various experimental facilities like greenhouses, fields, phenotyping networks, or natural sites. It can be done using many different devices from hand measurements to high throughput means. The resulting complex and heterogeneous datasets include all the environment and phenotypic variable values at each relevant scale (plant, micro plot,…) and very importantly the identification of the phenotyped germplasm,* i.e.,* the plant material being experimented. In addition, there are often relationships between levels,* i.e.,* physical scales, inside datasets and between different datasets. The resulting rich wealth of data is usually formatted in a very heterogeneous manner and is difficult to integrate automatically.

Phenotyping experiments are expensive and are not exactly reproducible since the environmental conditions are difficult if not impossible to completely control. Furthermore, most traits are highly dependent on genotype by environment interactions, which increases again the uniqueness and the value of the data collected to describe environmental conditions and resources available to the plants during their lifecycle. Hence, being able to reuse phenotyping data to carry out large meta-analysis would allow better deciphering the genetic architecture of traits across environments. It could help the prediction of genotype performances in the context of the climate change adaptations. An example is the use of data series collected over centuries that have demonstrated or supported the modelling of the impact of climate change on crops [3, 4]. In this context, long-term data management following the Findable, Accessible, Interoperable, and Reusable (FAIR) principles [5] is among the main challenges of modern phenomics. There are two answers to this challenge: data standardization and data integration.

Several initiatives are developing tools for standardizing phenotyping data description. The Minimal Information About Plant Phenotyping Experiment (MIAPPE, www.miappe.org) [6, 7] defines the set of information necessary to enable data reuse. This includes the objective of the experiment, the authors, location, and timing, as well as the minimal description of the* observation units*,* i.e.,* the objects being measured and assayed, including the plant material identification and the traits with their measurement protocols. The latter are formalized through the Crop Ontology (CO, www.cropontology.org) [8] which states that all observations and measurements are done through an* observation variable* which is defined by three components: (i) the targeted trait (phenotypic or environmental), (ii) the method of measurement or observation, and (iii) its scale or unit. A trait can be formalized as the association of an observed entity like a part of the plant (e.g., leaf, grain, and stem) and an attribute or quality to be measured or observed (colour, weight, and height) [9]. The method can be a phenotyping protocol or a statistical computation and can include cross references to method books or software. A new variable is created each time a new method or a new scale or unit is added to an existing trait. The Crop Ontology provides a collaborative platform to a growing number of crop communities to develop a series of species-specific ontologies. The Planteome project (http://planteome.org/) links through a semantic mapping these species-specific ontologies to a set of reference plant species-neutral ontologies including the reference Trait Ontology (TO) and the Plant Ontology (PO) [10]. This annotation process adds a generic trait above the crop-specific traits [11]. This helps to connect crop phenotyping data to genomic data across species. Besides, through the mapping, CO inherits the ontological structure of TO and can be used for building an ontology optimized for data sharing and integration between crop research communities. Finally, the Breeding API (BrAPI, www.brapi.org) [12] is building a specification of web services to enable standard data exchange between information systems and tools. All these tools are facilitating data standardization and are now widely adopted by the international plant community [13–15].

Data integration relies on datasets and data repositories interoperability and links different datasets together [16] in order to avoid data silos. It is achieved by following the Linked Data principles [17] and in particular by defining and identifying the key resources,* i.e.,* the key “things” in the Web Ontology Language (OWL) sense, that acts as interoperability pivot by linking one dataset to another. These interoperability pivots, shared between datasets, enable the construction of datalinks and must be unambiguously identified and curated in each data repository. Pivot identifiers must be shared among repositories to enable data interoperability and build a working information system federation. Indeed, phenotyping experiments can be carried out for a wide range of scientific objectives (*e.g.,* study of the impact of climate change, study of the genetic architecture of traits) with different types of underlying analyses that impact the nature of datasets. The consistency of the datasets is ensured through the integration of the data collected from the different experiments, which is achieved by building links between some clearly described and identified pivots. A common example is the integration of genotyping and phenotyping datasets obtained with the same panel of individuals in distinct experiments in order to search for marker-trait associations. In this case, individuals of the panel in each dataset provide the pivot required to enable interdataset integration. Other examples of interoperability pivot are the Global Positioning System (GPS) localization of plants (*e.g.,* integration of climatic and phenotyping datasets) or the observation variables (*e.g., *integration of several phenotyping datasets).

When managing and therefore integrating research data in any Phenotype Information System, the objectives of the data services to be provided must be considered. For instance, the MaizeGDB [18] database gives access to phenotypic data in the context of functional genomics studies by offering very elaborated phenotype without experimentation environment data. Genomes To Fields (https://www.genomes2fields.org [19]) and the Triticeae Toolbox [20] offer more trial centric portals for, respectively, the US maize and the US Triticeae communities. All these repositories allow sharing and publishing curated datasets but neither data discovery nor multitrial data integration. There are also a number of trial-centric databases whose objectives are to capture all the steps of the data production of platforms, like PhenopsisDB [21], the Integrated Breeding Platform (IBP, https://www.integratedbreeding.net), Phenomics Ontology Driven Database (PODD) [22], or the Phenomic Hybrid Information System of the Phenome-Emphasis (https://www.phenome-emphasis.fr/) infrastructure (PHIS, http://www.phis.inra.fr) [23]. This latter database, PHIS, is specifically designed for addressing the challenges of data acquisition in high throughput phenotyping platforms.

GnpIS [24] (http://urgi.versailles.inra.fr/gnpis) is an international information system that links phenomic, genetic, and genomic data (see examples in [25, 26]) for plant and their pathogens. It is the French National Institute for Agricultural Research (INRA) phenotyping archive which has been designed to publish and integrate standardized data from phenotyping trials carried out in natural sites, field, or controlled environments, with observations at different physical scales like groups of plants, single plants, single organs, or tissues. It gives access to standardized data and enables the development of federations of repositories.

## 2. Material and Methods

The GnpIS software component dedicated to phenotyping, named GnpIS-Ephesis, is based on a four layers' architecture, described in the result section: storage, data discovery, query, and web interface.

The storage layer of GnpIS-Ephesis is implemented in PostgreSQL 9.6 running in a 2-core 4 Gb Virtual Machine plus file-system access through simple HTTP GET requests for direct file download.

The query layer is based on Elasticsearch 2.3 running on Java 7 in two 8-core 16Gb RAM Virtual Machines. It allows precise, field-by-field, data querying and processing. Its native Representational State Transfer (REST) API is hidden behind a service business layer for security and ease of querying. Its API is queried either by Google Web Toolkit Remote Procedure Call (GWT RPC) or by a REST Web Service API. This Web service layer is written in Java/JEE using Jersey 1.18+ and Spring 2.5. The Extract Transform Load (ETL) tools allowing for feeding the query layer from the storage layer are written in scripted PostgreSQL specific JSON-SQL queries orchestrated by a Shell tool suite.

The data integration and insertion toolbox is developed with the Talend Open Studio (http://www.talend.com/) Extract Transform Load (ETL) tool version 6, plus some Shell and Python scripts.

The ontology repository is based on a public Gitlab project running on the INRA forge (https://forgemia.inra.fr/urgi-is/ontologies) which allows versioning of the ontologies plus a graphical widget giving access to their last versions. The Ontology Widget (https://github.com/gnpis/trait-ontology-widget) is written in JavaScript and uses the JQuery (https://jquery.com/) and JStree (https://www.jstree.com/) libraries.

The Web interfaces are running on a Tomcat 7 instance using Java 7 in a dedicated Virtual Machine with 2 cores and 16Gb of RAM. They are developed in Java 7 using the GWT framework. The geographic map overview is powered by Leaflet (https://leafletjs.com/) with OpenStreetMap (https://www.openstreetmap.org/) as map backend.

The web user interfaces are open source under BSD3 license and available upon request. The database model of the storage layer is under a proprietary license and is protected by deposits in at the European program deposit agency (Agence de Protection des Programmes).

## 3. Results

GnpIS is a repository for phenotyping experiments,* i.e., Trials*, at various physical and temporal scales. It has been developed within the GnpIS-Ephesis project which gave its name to the software modules of GnpIS dedicated to phenotyping. The experimental data may be associated with measurement time, hence creating time series. Data can be raw or computed, organized in textual data matrices of physical measures possibly derived from sensors, phenological observations, or concentrations for a few dozens of biochemical components. Those data matrices are inserted in the storage database together with additional information like factors, cofactors, timing, location, and other trial parameters description. In some cases, such as dense time series with up to hundreds of measures, multispectral images, or Near Infrared Spectrometry (NIRS) spectra, the data can be stored as files (with a size limit of few Gb by Trial) or can be cross-referenced to specialized platform information systems. It is designed to allow data access either by full experiment or by aggregating data across several experiments. It also allows the linking of phenotypic data with genetic and genomic data for Quantitative Trait Loci (QTLs), Genome Wide Association Studies (GWAS), and gene annotations published in GnpIS.

GnpIS currently stores data for the French National Institute for Agricultural Research (INRA) and its national and international partners. It is the official repository of the International Wheat Genome Sequencing Consortium [25] and it is included in emerging international federations of information systems in the frame of the Elixir plant community (https://www.elixir-europe.org/communities/plant-sciences), the French node of the Emphasis European infrastructure for plant phenotyping (https://www.phenome-emphasis.fr/), and the global WheatIS of the Wheat Initiative (www.wheatis.org). Public and private data from phenotyping experiments are currently available for wheat, grape, maize, tomato, rapeseed, pea, and forest and fruit trees (Table 1).

This high level of integration and interoperability relies on the proper identification of interoperability pivots: mainly the plant material or germplasm and the observation variables (mandatory) and to a lesser extent the location and timing.

### 3.1. GnpIS Phenotyping Data Model

**Figure 1:**
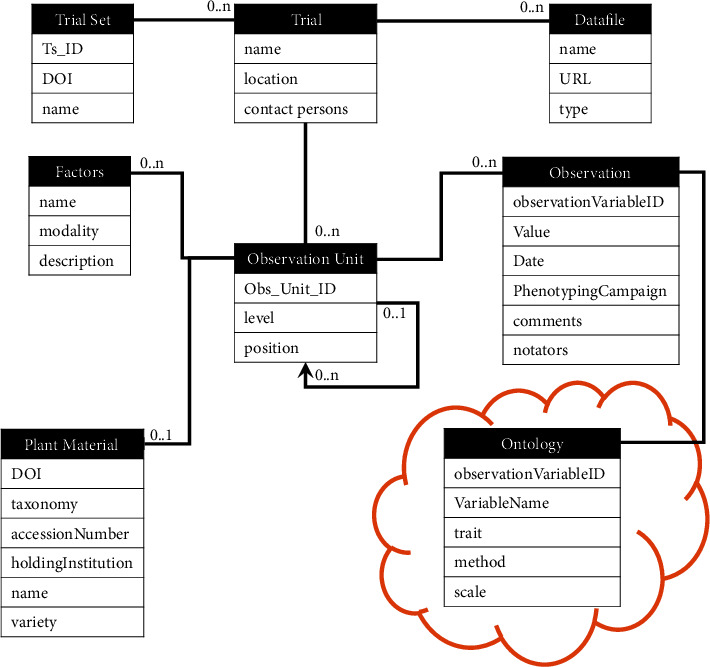
GnpIS phenotyping conceptual data model. A trial (MIAPPE Study) corresponds to a single experiment in one location, possibly over several years. Multilocation trials are represented as Trial Sets. Data from phenotyping experiments are organized around two main entities: Observation Unit and Observation. The Observation Unit represents any level at which the plant material has been observed. The levels hierarchy is stored through a recursive link. The Observation Unit is linked to a single Plant Material and a single modality of each Factor. Each Observation has an observationVariableId, taken from a relevant Ontology, a Value (numeric, date, file URL,…), a Date, and a PhenotypingCampaign, which acts as a tag to group several measurements within a trial like a year (2007), a group of years (1956-2012), or a season (2012 spring). Variables are described using ontologies that follow the Crop Ontology model, for both phenotypic and environment measurements. The Ontology is managed as an external source linked to the observation through linked data principles rather than directly integrated in the dataset.

Phenotyping data is handled in GnpIS through the GnpIS-Ephesis conceptual data model (Figure 1). It has been designed in close collaboration with field scientists, experts in plant phenomics, geneticists, and breeders, many of them being particularly interested in deciphering genotype by environment interactions. It has been designed for flexibility, to allow both the retrieval of individual datasets and the combination of different subsets for meta-analysis. It relies on three main components: (i) the main dataset containing the description of the trial and the* observation units *(Figure 1) as well as the observation values, (ii) the* observation variables,* and (iii) the identification of plant material assayed. Those three components act as independent but linked subdatasets. This structure allows to update the description of the plant material or of the variables without affecting the main phenotyping dataset. The GnpIS-Ephesis data model is continuously improved to remain compliant with the MIAPPE [6] standard evolutions. Datasets can be published along with a Digital Object Identifier (DOI) [27] which provides authorship, reuse license, and citability.

#### 3.1.1. Trial, Trial Set, Observation Unit, and Observation

**Figure 2:**
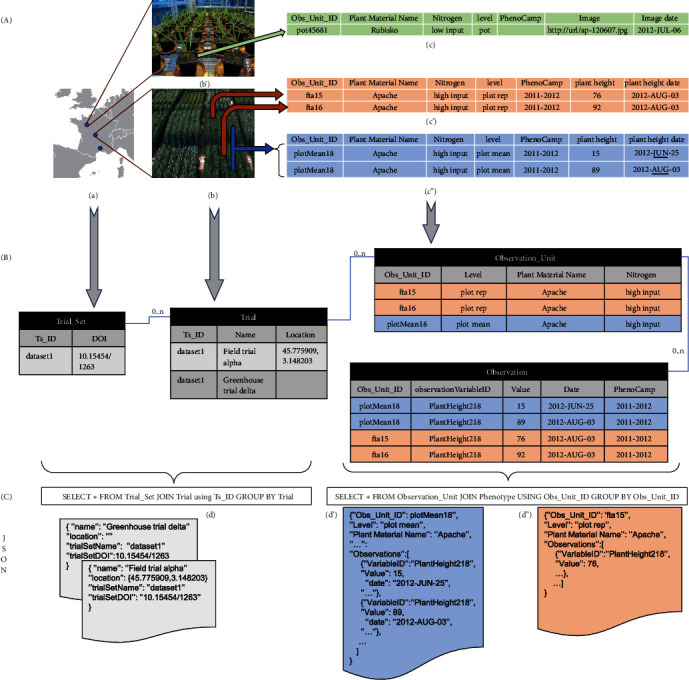
GnpIS-Ephesis data retrieval. (A) Experimental data input for GnpIS. (B) Summary of the data model captured in the storage layer of GnpIS. A list of Trials forms a Trial Set (a). A Trial Set can be either a phenotyping network or a consolidated dataset. A Trial can be either a field trial (b) or a greenhouse trial, including automated ones (b'). Phenotyping observations and measures are made on the Observation Unit which represent different scales, or levels, of observation:* e.g.,* a pot in a greenhouse (c), a single microplot (c'), or a mean of all microplots of the same plant variety (c”). Note that (c) represent only one pot, (c') represent two microplots (fta15, fta16). (c”) displays a time series on the Observation Unit plotMean18; each value is a mean of fta15 and fta16. The concept of Phenotyping Campaign (PhenoCamp) allows the grouping of observations within a Trial; it is used for perennial plant to group observations by years and for network carrying multilocal and multiyear trials in annual plants to easily filter data from all trials conducted on a given year. The treatment (*Nitrogen* column here) is an experimental factor whose effect is under study. The Phenotype Values can be simple numeric values (c' & c”), files with a URL or URI pointing to a file repository (c), or phenological dates. Note that, to improve the clarity of the figure, the real ontology variableID has been replaced here with PlantHeight218. The query layer (C) is designed for fast answers and aggregates multiple entities in a few JSON documents, like the Trial (d), an extension of the Breeding API Study, and the ObservationUnit (d' and d”). They are directly generated from the storage layer through SQL queries using PostgreSQL functions. There is one Trial document by trial and the Trial Set information are duplicated in each of them. The (d') JSON document clearly shows how the Phenotypes for *«*plotMean18*»* are aggregated as a time series in a single observation unit.

Figure 2 shows a typical phenotyping dataset and how it is integrated in GnpIS through four main concepts: Trial Set, Trial, Observation Unit, and Observation.

The* Trial* and* Trial Set* handle most of the experiment metadata. A trial is an experiment under field or controlled conditions (greenhouse, culture chamber…), in a single location and possibly on multiple years. This allows for handling series of yearly observations for perennial plants, possibly over several decades. Note that, in this case, the plant material list is stable from one year to another. Multilocation experimental networks are modelled as a Trial Set with one Trial per location. There is a good mapping with MIAPPE v1.1 (www.miappe.org and more precisely https://github.com/MIAPPE/MIAPPE/tree/master/MIAPPE_Checklist-Data-Model-v1.1) where the Trial Set corresponds to the* Investigation* and the Trial to the* Study*.

The* Observation Unit* in GnpIS and MIAPPE v1.1 is the object,* i.e., *the scale or level, on which the measurements or observations are done (Figure 1; example in Figure 2(B)). It is possible to describe different scales in the same experiment. The scale name is ontology driven, but there is no recommended level ontology at the time of writing. Therefore, we have our own controlled vocabulary (e.g., micro plot, plant, and pot) which can grow upon requests from our data submitters. Some details of the scientific design are stored as Observation Unit fields, alongside the unit position and all the experimental factors. The Observation Unit stores the combination of the mandatory genotype factor (Plant Material below) with optional treatment factors (*e.g.,* Cultural practices, Irrigation, Nitrogen,…). Each treatment factor has a list of two or more possible values or modalities, (e.g.,* high input *and* low input* for the factor* Nitrogen* on Figure 2(B)). Each Observation Unit is associated with only one modality of a given factor. For instance, a Trial can combine a factor* Nitrogen*, with modalities* low input* and* high input*, plus a factor* Water* with* no watering* and* watering* modalities. Each observation unit allows for observing the behavior of a single genotype under a combination of one modality from each of the two factors.

The* Observation* is ontology driven, with all metadata stored following the Crop Ontology framework [8]. It allows for storing Phenotype or Environment measures. The Observations consists in triplets formed by an Observation Variable described below (e.g., yield in q/ha, plant height in cm, rust score,…), a value (the measure), and an optional date (Figures 1 and 2). Additional metadata can be stored either as linked files, for cultural practice or soil analysis reports, for instance, or as events and observation like lodging scores or hail date. The Observation Unit and Observation data model have been inspired by approaches like The Extensible Observation Ontology (OBOE) [28, 29] and the GMOD Chado Genomic Feature [30]. In MIAPPE, the observations are stored in the data file.

#### 3.1.2. Plant Material

The phenotyped plant material, or germplasm, is the main interoperability pivot in GnpIS. Its correct identification varies depending on the context, but this problem has been discussed for several decades now and is addressed by an internationally recognized data standard, the Multi Crop Passport Descriptors (MCPD) [31]. Its importance and possible related issues are described in the study by Adam-Blondon et al. [13].

GnpIS is MCPD compliant and slightly extends it to fit the needs of its communities of users. In particular, our system handles experimental material that is not conserved in Genbanks as well as the concept of* Lot* which is a group of seeds or plant derived from a single accession. The identification of accessions in the MCPD relies on a triplet of information: the accession number, the holding institution, and the genus plus optional species. The Accession Number is the actual identifier of the plant material and must be unique in the holding institution and genus namespace. This triplet is now completed in GnpIS by a permanent unique identifier through a DOI or an URI (Unified Resource Identifier), as recommended by the FAO (International Treaty on Plant Genetic Resources citation). Those permanent unique identifiers are unique at the scale of the World Wide Web.

This allows for storing a comprehensive description of the plant material at different levels: identification of the accession of a germplasm collection and of a derived seed lot used in an experiment and the corresponding variety name. For instance, in a Zea maize trial, the variety B73 would have been provided by the INRA maize collection under the accession identifier B73_inra and the B73_inra_SMH08 seed lot was experimented.

#### 3.1.3. Observation Variable

The second important interoperability pivot is the* Observation Variable,* formalized by the Crop Ontology as three terms that describe (i) the phenotypic or environmental trait, (ii) the method used for the observation or measurement, and (iii) the unit or scale used for this observation [32]. The variable annotates the actual measurement,* i.e.,* Observation, made during the trial. To support FAIR data, the* Observation Variable* must be fully described and the three terms must be agreed and shared within the relevant crop communities.

### 3.2. Software Architecture

**Figure 3:**
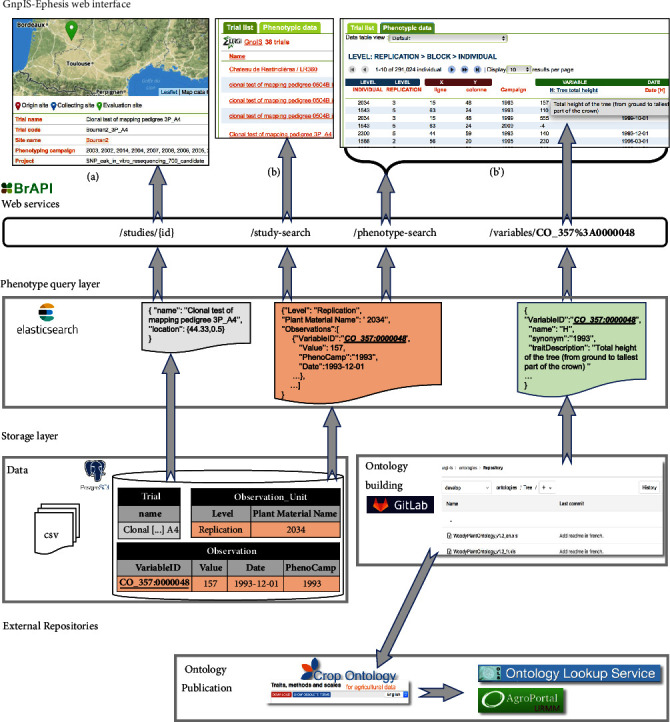
GnpIS-Ephesis Information System architecture. The storage layers uncouple ontology and data. Only the observation variables ID (for instance, CO_357:0000048 for Plant height) are stored in the database. As a consequence, Observation Variable ID are never suppressed from the ontology but they can be marked as obsolete or deprecated. The full description of the variables is stored in a Cropontology.org Trait Dictionary V5 Excel file versioned in the INRA gitlab (https://forgemia.inra.fr/urgi-is/ontologies). The query layers index the storage layer with dedicated ETLs. GnpIS uses a BrAPI compliant web service layer. The web interface provides the user with a web form, not shown here, that allows querying of the main pivots. A Trial card (a) displays all information for each individual trial and allows the download of trial specific data files. The result page displays an overview of the Trials (b) and of the phenotypic data (b'). Note that a single web page (result page (b) on this example) uses multiple web services (study-search, phenotype-search, and variables here).

An overview of the software architecture of GnpIS-Ephesis is given in Figure 3. Its originality is to isolate the long-term storage of the data from the query layer, which is specific to the current web services and user interfaces. Furthermore, the user interface and the query layer are connected through web services and inspired by the microservice architecture. The storage layer consists of (i) a relational database which implements the conceptual data model and stores the two-dimensional data matrices and (ii) a file repository that stores data files such as images, global description of cultural practices, soil characteristics, NIRS results, and ontologies. The storage layer uncouples components of the phenotyping datasets to ease data curation and update. This is fully implemented for observation variables where datasets are stored in the database and ontologies in the file repository as seen in Figure 3. This allows for updating the ontologies without interfering with the* Trials* storage.

The storage layer relational model is almost fully normalized (in the third normal form) which makes it efficient for storing consistent data on the long term but difficult to optimize for fast querying. Indeed, filtering the data or rebuilding the data matrix for export involves SQL joins between the Observation Unit table (more than 360 000 rows in 2018) and the Phenotype table (near 4 million rows in 2018), plus most of the other tables of the model. This join is costly even with fine-tuned indices such as composite indices or programmatic optimizations,* i.e*., using several light queries rather than one expensive query. To address this problem, we have explored data denormalization with a pure SQL approach. This proved to be efficient for the expected volumes but was not flexible enough to handle heterogeneous phenotyping data, in particular with respect to the varying width of the data matrices. Furthermore, NoSQL systems allow much easier horizontal scaling to cope with data volume increases.

The phenotype query layer was therefore introduced as a document-oriented NoSQL system based on Elasticsearch. Trial and Observation Unit documents are aggregating all the data necessary for querying, filtering, displaying, and exporting whole datasets. This document structure is based on the denormalization of the first normal form [33] by aggregating several objects in a single document. For instance, the trial document includes all the information about locations (coordinates, names,…), plant material, and authorship. This simple aggregation is completed by a nesting of data graphs in the documents which can be seen in the Observation Unit document where all observations are listed as objects including value, variable, time, and metadata (Figure 2). Thus, no costly joins are needed between Observation Unit and Observation, well known problems like the select n+1 are avoided and the response time is below one second. GnpIS JSON Documents have been modeled in collaboration with the Breeding API (BrAPI) consortium [12]. GnpIS has contributed to BrAPI with the Observation Unit model and we have adopted the BrAPI Study, Observation Variable, and Germplasm documents which are based on shared standards.

### 3.3. Web User Interface

GnpIS provides phenotyping data discovery capabilities and data aggregation among several datasets. The dedicated query form, available in the phenotyping section of GnpIS (https://urgi.versailles.inra.fr/gnpis/), is based on three tabs: (i) “Genotype” for filtering the plant material by species, genetic panel, and collections, (ii) “Observation variables” that allows variables selection using a Breeding API compliant open source widget (https://github.com/gnpis/trait-ontology-widget), and (iii) “Trial” that contains filters for general metadata like the Phenotyping Campaign,* i.e., *Year, the location, the datasets list, or project filtering. The trait-ontology-widget (Figure 3) provides a biologist friendly tree navigation and keyword search in the ontologies and displays the full details of each variable. It is specific to the Crop Ontology model and therefore relies on the BrAPI observation variables Web Services rather than a generic ontology server like the Ontology Lookup Service. The selected variables are used to filter the phenotypic data search. It can easily be integrated in any system and is available in the BrAPI Application Showcase (BrAPPs, https://www.brapi.org/brapps.php). Note that the search filters apply not only to the Trials but also to the actual data. In other words, when filtering with a specific variety, we will preview only the trials using this variety, but also only the measurement made on this particular variety. This cross-tab filtering is useful to guide users in the search criterion selection steps.

**Figure 4:**
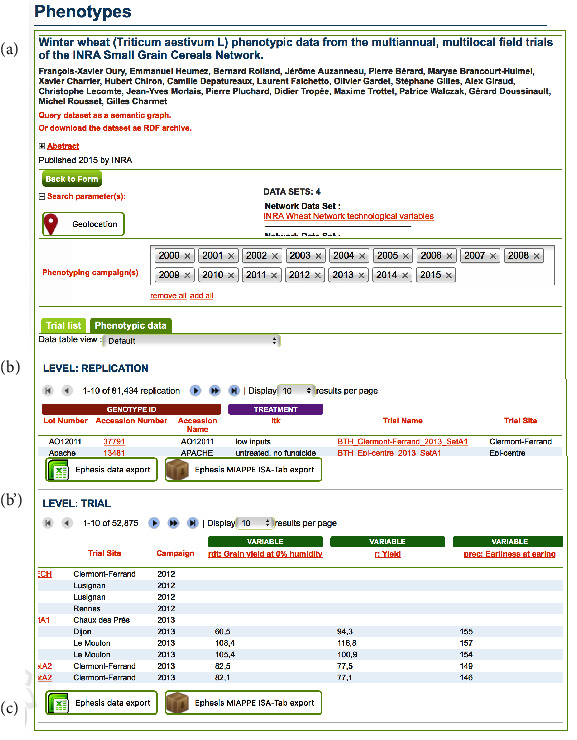
Overview of the main GnpIS-Ephesis result page. The figure illustrates the main features presented in the interface with the exception of the interactive geographic map, which is not displayed for simplification. (a) When a DOI has been associated with a trial set, it is used to display all the authorship metadata fetched directly from doi.org. The time of the experiment can be filtered through the list of phenotyping campaigns. (c) The result dataset can be downloaded for further use in two formats: a.*csv* file and a MIAPPE compliant machine-readable ISA Tab zip archive which provides both the data files and all the metadata associated. Several levels of data are displayed (b & b'). Each line of the data matrices corresponds to one Observation Unit and shows the combination of genotype and cultural practice factors (itk on the screenshot).

The result page (Figure 4) provides an overview of trials location through an interactive map. The list of selected trials is displayed in the “Trial list” tab. On the “Phenotypic data” tab, the data from several trials can be previewed with one data matrix by level. Each line of a matrix corresponds to one observation unit. It includes most of the metadata necessary for traceability and reliable data analysis.

From the result page, several cards can be accessed to give synthetic overview of key objects, the main one being the Trial and the Accession. The trial card displays all the MIAPPE metadata, plus a free list of key value pairs for additional trial information. The accession card displays all MCPD metadata, the genealogy, primary descriptors (trial independent phenotypic values like the shape of the fruit), pictures, panels, and collections.

### 3.4. Web Services Open API

GnpIS allows data access through Open API (https://www.openapis.org/) compliant web services implementing in particular the Phenotype related sections of the Breeding API, including Germplasm, Study, Location, Observation Variables, and Phenotypes. GnpIS includes BrAPI clients and a publicly available server-side implementation on top of the query layer. A swagger interface provides documentation and a test bench (https://urgi.versailles.inra.fr/Tools/Web-services).

### 3.5. Data Management

GnpIS data publication and integration process includes both a data review step by data managers and an automated validation step to ensure a good balance between data submission ease and data quality. It starts by filling a tabular exchange format available through the web application. This format is the result of several years of collaboration with biology experts including geneticists, agronomists, genotype by environment specialists, researchers, and experimentation managers all working on annual or perennial plant, including forest trees. This exchange format has been designed to be both human and machine readable. This allows data validation and curation by data producers as well as efficient and reliable parsing before database insertion. When submitting a dataset, the users must first consolidate their interoperability pivots. The plant material list must be submitted with minimal information necessary for its identification and GnpIS data managers work in close collaboration with the curators of the INRA genebank collections.

The observation variables are handled through the workflow developed with the Crop Ontology Trait Dictionary exchange format v5 (TDv5), with the assistance of GnpIS data managers. They can be either chosen within an existing ontology, added to an existing ontology, or listed in a new dedicated one. Indeed, whole comprehensive new ontologies have been created, like for grapes (Vitis Ontology) or Forest trees (Woody Plants Ontology) (Table 2). As seen in Figure 3, the ontologies are managed and versioned in the INRA GitLab in Crop Ontology TDv5 format, before being integrated within the data layer. Some of them are also being published on the Agroportal [34] and on the Crop Ontology portal which is synchronized with the EBI Ontology Lookup Service [35]. It has sometimes been necessary to create some new parallel ontologies for species which were already present on the Crop Ontology portal. It indeed facilitates the capture of the information about their phenotyping variables in large consortia with a history of data sharing practices. This is the case for the Wheat INRA Phenotype Ontology (WIPO) that shares many traits with the CIMMYT Wheat Crop Ontology (published on the Crop Ontology portal) but lists measurement methods specific of their respective user communities. The merging of those two ontologies is in progress. More than ten ontologies are currently used in GnpIS (Table 2).

A data stewardship service to support users in their submission and curation work is offered allowing so far the publication of more than a thousand trials (Table 2). Fully formatted GnpIS exchange format files are submitted, validated, and inserted using the GnpIS toolbox. Dedicated workflows can also be developed collaboratively.

## 4. Discussion

The phenotyping data life cycle main steps are data collection, quality control including curation and cleaning, analysis, publication, sharing, and finally reuse. GnpIS mainly supports the three last steps while, for instance, the recently published PHIS [23] supports mainly the first three. Experimentation datasets usually include three types of data: (i) raw untransformed data (images, multispectral images, NIRS, frequencies, etc…) which are transformed into (ii) raw transformed data (in International System units, including dates) and finally (iii) elaborated or derived data (stress resistance, biomass, leaf area index, etc.). Depending on the needs, data of the second and third types can be directly managed in GnpIS whose data model has been designed to handle both field and greenhouse experimental data.

GnpIS focuses on interoperability and integration capabilities through the usage of MIAPPE, the Breeding API, and the Crop Ontology standards. The system is therefore very versatile and can be used to integrate and consolidate datasets suitable for genetics studies, trait diversity studies in genetic resources, or modeling approaches in physiology.

### 4.1. How FAIR Is GnpIS?

Currently, phenotyping data in GnpIS implements mainly the “FAIR for the human” as described in the study by Wilkinson et al. [36]. It is well advanced and allows a good traceability of the data acquisition methods, of its transformation, and experimentation factors. But that information still needs to be expressed with more advanced formalisms to enable FAIR machine readability and to improve the quality of the metadata. Indeed, enabling FAIRness for machines would in particular imply the use of semantic formats,* i.e.,* Resource Description Framework (RDF) and JSON-LD. It is a complex objective that is not only technical but would require an evaluation of the FAIRness of each of the datasets integrated in GnpIS, which is not yet done. In addition, the linked data principles [17] state that every resource must be correctly identified with an HTTP URI, described in RDF, and linked to other resources. This has been partially implemented in GnpIS: the interoperability pivots (Variables, Accessions, and Datasets) are linked to other resources with permanent unique IDs but only the accessions and some datasets have DOIs or URIs. Nonetheless, with the right namespace, GnpIS IDs are unique at the scale of the World Wide Web and therefore provide a strong basis for future full enabling of linked data in GnpIS. The interoperability of GnpIS with other databases is ensured by REST Open APIs, and especially the increasingly adopted Breeding API. REST is well integrated with the current web application development ecosystem. As a consequence, RDF is not planned to be used directly as the main medium for linked data in GnpIS, which will rather be enabled through extension of those APIs using the JSON-LD semantic format, hence enabling the conversion to RDF. A proof of concept has been realized with a Wheat dataset available in a dedicated triple store and as a downloadable RDF file (see link to data in the DOI of [37]).

Findability of the datasets by users is enabled through indexing rich metadata and fast querying mechanisms. Accessibility is guaranteed by long-term storage associated with open technologies (HTTP REST) and format (CSV, JSON, and ISATab). The license is by default Creative Commons (CC-BY 4) and can be modified through a DOI associated with specific datasets.

Interoperability in GnpIS also relies on data curation and integration aiming at the unambiguous identification of the pivot data and the use of standard formats for metadata descriptions and vocabularies, which is a costly effort [38]. In our experience, the most difficult points are the correct identification of the plant material and the development of the appropriate Crop Ontology variable list when it does not exist yet. This curation process is greatly eased by the uncoupling of the datasets and the ontologies which allow seamless updates of the variable ontologies. Indeed, upgrading an ontology version, or switching back to a previous version in case of problems, can be done in less than an hour by a data manager. The Crop Ontology community is also working on easing the process of building and enriching ontologies from information systems like Cassavabase [39] which provides a web form for creating or requesting new variables.

The use of the CO approach and trait dictionary format to submit* Observation Variables* in GnpIS has two objectives. The first one is to guide and capture agreements within a research network on measurement methods which allows consistent data collection and analysis. The originality of the Crop Ontology approach [40] is to build a set of species specific, or clade-specific, variable ontologies, rather than building a global variable ontology, which would be difficult if not irrelevant. Therefore, the second objective is to focus on a better standardized list of traits and to let communities freely create methods and variables adapted to their research. This work has begun within the Planteome initiative, and could be extended by publishing common Trait lists. To ease this process, we are considering maintaining two sets of ontologies for some species, one to address the specific needs of GnpIS communities and to act as a clearing house for variable curation and validation and the other which is much broader and therefore published on references portals. With this pragmatic approach, the FAIRness of the datasets is ensured either by annotating with existing ontologies, published in Crop Ontology, or by creating* ad hoc* ontologies following the proven CO model.

Particular* Observation Variables* use cases needed some adaptations of the recommendations of the Crop Ontology while keeping semantic interoperability. A good example are complex variables, elaborated by combining several variables like, for instance, measurement of plant height at flowering (combination of flowering time and plant height time series) or green Berry pH and mature Berry pH (combination of berry composition with phenology). In those examples, we are dealing with classical trait/method/scale variables combined with a development stage or a treatment duration. Creating the variables covering all the needed combinations would lead to ontologies with several thousands of variables. GnpIS proposes to create complex variables specific of the trial and which are not listed as such in the Crop Ontology. Each of those specific variables are annotated by a crop ontology variable, hence linking them to reference variables. For instance, the variable* Canker lesion length* (CO_357:0000088) annotates two local variables, Bacterial canker lesion length 1 or 2 years after inoculation (Canker_length.2 and Canker_length.1). This example can be found with the Trial Code “POP2-Orleans-chancre”. This way, any variable necessary for a given experiment can be freely created as long as it is linked to a variable existing in a crop ontology. In the future, those specific variables could be simple text description annotated with IDs taken from several reference ontologies (e.g., Plant Environmental Condition Ontology for the treatment part, Plant Ontology for the growth stage part,…).

Curation of the plant material identifiers is more difficult to achieve. Indeed, while the MCPD standard provides identification principles, their application is community-based and cannot be automated for the moment. Currently, the plant material ID curation is a prerequisite for each dataset integration and publication in collaboration with the data providers. Once achieved, GnpIS associates with each accession a DOI generated by INRA to ensure a good traceability of the plant material and an unambiguous identification across any federation of information systems. This curation process, however, can introduce a delay in data publication.

Reusability in GnpIS varies from one phenotype dataset to the other. Data is generally available in easily parsable standard open formats: OpenAPI (BrAPI), JSON, and MIAPPE compliant Isa Tab or csv. They are currently being improved to better handle traceability of environment parameters and field practices. This type of data can currently be handled in GnpIS through variables like lodging or hail storm dates, comments on each variable or files describing field practices attached to the Trial. There is, however, no clear standard way yet proposed in GnpIS for this type of data. Since they are very important on the long term for meta-analysis, their submission should be facilitated in the future through a full upgrade of GnpIS to MIAPPE v1.1. Finally, the documentation of the provenance of the dataset, including measurement methods and data processing, is only partial and varies too much. The use of dedicated systems like the Phenotyping Hybrid Information System (PHIS) by the data producers would certainly facilitate the capture of all the metadata and their MIAPPE compliant publication in GnpIS.

### 4.2. Enhancing Community Building around Open Data in Plant Science

Making data FAIR is necessary to enhance knowledge development and innovation but has an important cost as it requires time of different types of experts to standardize the data (experts in standards maintaining registries and often tools facilitating their use, experts in the specific type of data considered, and computer engineers maintaining the repositories). It is therefore important to build international communities of practice around suites of tools that facilitate the generation of linked data and ensure a better sustainability of these tools. MIAPPE, BrAPI, and the Crop Ontology are good examples of such suites that are the products of a close collaboration between computer scientists and biologists from various communities at the global level. The importance of the implication of end users is well demonstrated in the collaboration with the Crop Ontology. Indeed, the biologist friendly framework built within this initiative and based on the CGIAR experience has been easily adopted by GnpIS and Elixir Plant communities. This greatly helped to improve the quality of our datasets and in turn will open collaborations with large initiatives in the domain of plant ontologies like Planteome or Agroportal.

The implementation of these standards in GnpIS together with data curation efforts in collaboration with the data producers have been instrumental to ensure GnpIS interoperability at a larger scale. Indeed, GnpIS is included in international data repositories federations including Elixir plant community, Emphasis (https://emphasis.plant-phenotyping.eu/), and the WheatIS. The use of common global standards focused on interoperability allows independent updates of the members of a federation and should enhance the sustainability of the tools built at the global level to support the federation and in the end of the whole federation.

## 5. Conclusion

GnpIS provides an archive for phenotyping experimental data compliant to FAIR principles in terms of data access, traceability of the metadata, and citability of the datasets. It applies open data recommendations promoted by several national and international infrastructures, scientific societies, and funding agencies. It also allows for integrating different sets of data to support different types of researches in the field of the adaptation to environment or to the impact of climate change. As there is no global archive for phenotyping data, GnpIS has been built to be integrated in several federations of information systems accessible through common data portals, the oldest one being the WheatIS portal. This has been possible thanks to the continuous implementation of the current standards recommended by the international community, hence facilitating interoperability between information systems and data integration and providing strong foundations for new federations.

## Figures and Tables

**Table 1:** GnpIS-Ephesis data summary in October 2018. Private data access is restricted to project's consortia. Note that an accession corresponds here to an entry in a genebank and therefore to the level at which the plant material is identified.

Genus	Trials		Years		Accessions		Variables	
	Public	Private	Public	Private	Public	Private	Public	Private
Abies		12		1		300		71

Betula		21		1		531		72

Brassica	5		1		69		88	

Fagus		24		1		610		72

Fraxinus		1		2		1		7

Hordeum		7		1		511		24

Juglans	3		43		150		45	

Miscanthus		4		2		171		34

Picea		19		1		475		70

Pinus	10	63	23	1	790	1633	50	72

Pisum		86		5		610		265

Populus	5	18	3	3	336	1958	17	79

Quercus	22	18	28	1	1416	464	103	72

Salix		1		2		553		7

Solanum	2		1		193		42	

Sorbus	16		12		142		8	

Taxus		10		1		267		73

Triticum	820	37	18	3	2947	950	76	238

Ulmus		1		2		2		7

Vitis	5		58		871		39	

Zea	1	3	1	2	336	1780	16	26

**Total**	**889**	**325**			**7250**	**10816**	**484**	**1189**

**Table 2:** List of the main ontologies currently used in GnpIS. Only those on which GnpIS data managers have invested significant curation effort are listed here. Other ontologies, specific of some datasets are available in the GnpIS ontology portal. Ontologies are available in several formats (Crop Ontology TDv5, BrAPI, OWL/SKOS) in one or several repositories including GnpIS Ontology portal (https://urgi.versailles.inra.fr/ontology), Crop Ontology (www.cropontology.org), Agroportal (http://agroportal.lirmm.fr) and EBI Ontology Lookup Service (https://www.ebi.ac.uk/ols).

Ontology	Number of variables	Availability	Community
Brassica	164	GnpIS Ontology Portal https://urgi.versailles.inra.fr/ontology#termIdentifier=CO_348 Crop Ontology http://www.cropontology.org/ontology/CO_348 AgroPortal http://agroportal.lirmm.fr/ontologies/CO_348 EBI OLS https://www.ebi.ac.uk/ols/ontologies/co_348	New ontology following crop ontology framework originally built for French (Rapsodyn) and UK (RIPR) national Rapeseed projects.

Maize	17	GnpIS Ontology Portal https://urgi.versailles.inra.fr/ontology#termIdentifier=GNPISO_4	Contribution to the CIMMYT crop ontology.

Miscanthus	76	GnpIS Ontology Portal https://urgi.versailles.inra.fr/ontology#termIdentifier=BFF	New ontology following the crop ontology framework originally built for french national projects on Miscanthus. New ontology following crop ontology framework.

Protein Crops	192	GnpIS Ontology Portal https://urgi.versailles.inra.fr/ontology#termIdentifier=CO_349	New ontology following the crop ontology framework originally built for French national projects on protein crops: Garpen Peas, Dry Peas.

Vitis	278	GnpIS Ontology Portal https://urgi.versailles.inra.fr/ontology#termIdentifier=CO_356 Cropontology http://www.cropontology.org/ontology/CO_356 OLS https://www.ebi.ac.uk/ols/ontologies/co_356	New ontology following the crop ontology framework, originally built for publishing the internationally heavily used OIV descriptors (http://www.oiv.int/en/technical-standards-and-documents/description-of-grape-varieties/oiv-descriptor-list-for-grape-varieties-and-vitis-species-2nd-edition) in a more machine readable format.

Walnut	45	GnpIS Ontology Portal https://doi.org/10.15454/AV5RT2	New ontology following the crop ontology framework.

Wheat (WIPO)	277	GnpIS Ontology Portal https://urgi.versailles.inra.fr/ontology#termIdentifier=WIPO	New ontology following the crop ontology framework originally built for French and European wheat and barley projects.

Woody Plant	427	GnpIS Ontology Portal https://urgi.versailles.inra.fr/ontology#termIdentifier=CO_357 Cropontology http://www.cropontology.org/ontology/CO_357 AgroPortal http://agroportal.lirmm.fr/ontologies/CO_357 OLS https://www.ebi.ac.uk/ols/ontologies/co_357	New ontology following the crop ontology framework originally built for French and European forest tree projects.

## Abbreviations and Definitions

MIAPPE: Minimum Information about a Plant Phenotyping Experiment. Specifications and data model for data description
BrAPI: Breeding API (Application Programming Interface). REST (Representational State Transfer) Web Service specification to enable standard data exchange between information systems and tools
Crop Ontology: Both (i) an Ontology framework and data model to describe an observation variable and (ii) a Collection of species specific Variable Ontologies
FAIR: Findable, Accessible, Interoperable, Reusable
Ontology term: One element of an ontology, with a name, a definition, and relations to other terms. It is the equivalent of “concept” in SKOS (Simple Knowledge Organization System)
Observation Variable: One element of a Crop Ontology. Composed of a triplet of terms: Trait, Method, Scale
Dataset (Trial set): A consistent phenotyping dataset includes one to many trials, with their data and description (MIAPPE Study)
Trial: A phenotyping experiment in a single location for one (annual plants) to multiple (perennial plants) years
Germplasm: The plant material being experimented, including its unambiguous identification
Observation Unit: The objects being measured. It combines experimental factors, germplasm, and observation at a given scale
Level (scale): Defines whether an observation has been made on a single plant, a group of plants (plot), a whole trial, or any other scale
Observation (Measure): Phenotype or Environment Observation consists in triplets including an Observation Variable, a value, and an optional date or timestamp
Interoperability pivot (Key Resource): Objects and terms that can be unambiguously identified and can be shared among datasets, hence allowing link building. For instance, germplasms shared between genotyping and phenotyping datasets
Unique IDs: Unambiguous identifiers that must be valid at the scale of the world wide web. They include a prefix to define the namespace and the actual ID. For instance, URI (Uniform Resource Identifier) and DOI (Digital Object Identifier)
Open science: Movement and policy to give access to scientific publication, data, samples, and software. It encompasses open data and open source software
Open data: Policy and means to access any data type. It is enabled by applying the FAIR data principles
Open source: Software licensing principles that ensures free redistribution and modification of tools and systems source code.
